# Effects of Modified Atmosphere Packaging on Quality Maintenance of *Pleurotus pulmonarius* Under Simulated Logistics Temperature Fluctuations

**DOI:** 10.3390/foods15132366

**Published:** 2026-07-03

**Authors:** Junzheng Sun, Mengjie Yang, Na Zheng, Shanshan Wei, Shibo Li, Mingyi Liu, Jie Yang, Kai Ye, Pufu Lai

**Affiliations:** 1 Institute of Food Science and Technology, Fujian Academy of Agricultural Sciences, Fuzhou 350003, China; sunjzll@163.com (J.S.); yummy2023yummy@163.com (M.Y.); 15208653292@163.com (N.Z.); weisshan2024@163.com (S.W.); zhli0607@163.com (S.L.); 12409019066@fafu.edu.cn (M.L.); jaymi0718@163.com (J.Y.); yk_0312@163.com (K.Y.); 2 National R&D Center for Edible Fungi Processing, Fuzhou 350003, China; 3 Key Laboratory of Subtropical Characteristic Fruits, Vegetables and Edible Fungi Processing (Co-Construction by Ministry and Province), Ministry of Agriculture and Rural Affairs, Fuzhou 350003, China; 4 College of Life Science, Fujian Agriculture and Forestry University, Fuzhou 350002, China; 5 College of Food Science, Fujian Agriculture and Forestry University, Fuzhou 350002, China

**Keywords:** MAP, *Pleurotus pulmonarius*, logistics temperature fluctuations, postharvest quality, membrane integrity

## Abstract

Fresh *Pleurotus pulmonarius* is highly perishable during logistics because of its high water content, active respiration, and susceptibility to oxidative damage and membrane deterioration. This study optimized modified atmosphere packaging (MAP) conditions and evaluated their effects on postharvest quality and membrane lipid stability under simulated logistics temperature fluctuations. Single-factor and orthogonal experiments were used to optimize the package gas composition, including O_2_ and CO_2_ concentrations, as well as the packaging film. The selected MAP treatment (5% O_2_ + 20% CO_2_ with ethylene vinyl alcohol copolymer film) was compared with the control during 3 d of simulated logistics at 25 °C followed by 2 d of cold storage at 4 °C. Compared with the control, MAP maintained higher sensory quality, reduced weight loss and browning, and preserved total phenolic and flavonoid contents. It also inhibited O_2_^.^^−^ and malondialdehyde accumulation, enhanced superoxide dismutase, catalase, and ascorbate peroxidase activities, and delayed ascorbic acid and glutathione depletion. Moreover, MAP reduced membrane permeability, suppressed lipase, lipoxygenase, and phospholipase D activities, delayed phospholipid degradation, and maintained higher unsaturated fatty acid levels, U/S, and IUFA. These results indicate that MAP delays postharvest deterioration of *P. pulmonarius* during the 5-day simulated logistics and cold storage period, partly by maintaining ROS homeostasis and membrane lipid stability.

## 1. Introduction

*Pleurotus pulmonarius* is an edible mushroom valued for its tender texture, characteristic flavor, and nutritional and functional properties. Previous studies have shown that *P. pulmonarius* contains bioactive polysaccharides and other functional components with potential antioxidant, immunomodulatory, and health-promoting activities [1,2]. More broadly, edible mushrooms are recognized as nutritious foods rich in proteins, dietary fiber, minerals, vitamins, and bioactive compounds, making them important resources for both food and functional product development [3]. However, fresh mushrooms are highly perishable after harvest because of their high moisture content, soft tissue structure, and active metabolism. Postharvest *P. pulmonarius* is prone to quality deterioration during storage and distribution, including changes in water status, color, texture, sensory quality, and nutritional attributes [4]. Storage temperature and packaging conditions are critical external factors affecting mushroom quality, as inappropriate temperature control can accelerate respiration, microbial growth, and physiological senescence [5,6]. Therefore, effective postharvest preservation strategies are required to maintain the quality and marketability of fresh *P. pulmonarius* during logistics and short-term storage.

Temperature control and continuous temperature monitoring are essential for maintaining the postharvest quality of fresh agricultural products. Ideally, fresh products should be handled under a well-managed cold chain throughout storage and distribution. However, in practical logistics systems, especially when cold-chain facilities are incomplete or temperature control is insufficient, products may still experience temperature fluctuations during transportation and short-term distribution. In practical distribution systems, fresh products may experience changing temperature conditions because of incomplete cold chain facilities, insufficient temperature control, or limited logistics infrastructure. Laboratory-scale simulated logistics systems provide a practical approach for evaluating preservation strategies, as they can reproduce temperature changes during transportation while reducing the cost of real logistics testing. For example, simulated e-commerce logistics packaging has been used to evaluate microenvironmental changes and fruit quality variations during transportation [7]. For fresh mushrooms, logistics-related quality deterioration is often associated with accelerated respiration, water loss, microbial growth, oxidative stress, and membrane damage.

Modified atmosphere packaging (MAP) is an effective postharvest technology that regulates O_2_ and CO_2_ concentrations inside packages. Appropriate reduction of O_2_ and elevation of CO_2_ can suppress respiration, reduce metabolic consumption, inhibit aerobic microbial growth, and delay senescence. Previous studies have shown that high-CO_2_ controlled atmosphere storage can maintain postharvest quality and reduce browning in button mushrooms [8], while MAP film properties also affect the preservation quality of *Pleurotus* mushrooms [9]. However, unsuitable gas compositions may induce adverse effects; excessively low O_2_ and/or excessive CO_2_ can trigger anaerobic metabolism, resulting in off-odor formation and physiological injury [10]. Therefore, gas composition and packaging film must be optimized according to the physiological characteristics of each mushroom species.

Postharvest deterioration of edible fungi is closely related to reactive oxygen species (ROS) accumulation and membrane lipid degradation. Excessive ROS can damage cellular components, promote membrane lipid peroxidation, increase malondialdehyde (MDA) accumulation, and impair membrane integrity [11,12]. Antioxidant systems, including ROS-scavenging enzymes and non-enzymatic antioxidants, play important roles in maintaining cellular homeostasis during storage [11]. Meanwhile, membrane lipid degradation-related enzymes can promote phospholipid hydrolysis and fatty acid peroxidation, leading to membrane integrity loss and increased membrane permeability [12]. Previous studies have shown that preservation treatments can maintain postharvest quality by regulating ROS metabolism and membrane lipid metabolism in fresh edible fungi and other horticultural products [13,14]. Although edible mushrooms generally contain low total lipid levels, membrane lipids are essential structural components of cellular membranes rather than major storage reserves. Therefore, even small changes in phospholipid composition and fatty acid unsaturation may have important physiological consequences for membrane fluidity, permeability, compartmentalization, and cellular integrity. In fresh mushrooms, visible quality deterioration is mainly manifested as water loss, browning, texture softening, and spoilage symptoms; however, these changes are closely associated with cellular oxidative stress and membrane damage. Nevertheless, the mechanism by which MAP maintains *P. pulmonarius* quality under simulated logistics temperature fluctuations remains unclear.

Therefore, this study aimed to optimize MAP conditions for fresh *P. pulmonarius* under simulated logistics and subsequent cold storage. The effects of MAP on postharvest quality, antioxidant capacity, ROS metabolism, membrane permeability, phospholipid components, fatty acid composition, and membrane lipid degradation-related enzymes were investigated. This study provides theoretical support and practical guidance for improving the logistics quality and storage stability of fresh *P. pulmonarius*.

## 2. Materials and Methods

### 2.1. Materials and Treatments

High-quality ‘Jinxiu’ *P. pulmonarius* fruiting bodies cultivated in Luoyuan County, Fuzhou City, Fujian Province, China, were used as the experimental material. *P. pulmonarius* with uniform size, intact appearance, no visible disease, and no mechanical damage were harvested and transported to the laboratory on the same day. Surface impurities were removed before packaging.

The available film characteristics were as follows: EVOH film, thickness 0.025 mm and O_2_ transmission rate (OTR) 0.3 cm^3^·m^−2^·d^−1^; PET/PE film, thickness 0.06 mm and OTR 90 cm^3^·m^−2^·d^−1^; PA/PE film, thickness 0.06 mm and OTR 35 cm^3^·m^−2^·d^−1^; and PE film, thickness 0.06 mm and OTR 6000 cm^3^·m^−2^·d^−1^. These parameters were provided by the manufacturers. Polypropylene modified-atmosphere packaging boxes (22 cm × 13 cm × 4 cm) and ethylene vinyl alcohol copolymer (EVOH) co-extruded films were obtained from Jiangsu Dafeng Intelligent Technology Co., Ltd. (Nanjing, China). PET/PE, PA/PE, and PE films were obtained from Guangzhou Wanhe Packaging Materials Co., Ltd. (Guangzhou, China). All chemical reagents used for biochemical analyses were of analytical grade.

For the active MAP treatment, 100 g of fresh *P. pulmonarius* samples were placed in each polypropylene box, flushed with 5% O_2_ and 20% CO_2_, with N_2_ as the balance gas, and sealed with EVOH film using a modified-atmosphere packaging machine; this treatment was defined as the MAP group. The control samples were packaged in conventional polyethylene fresh-keeping bags without gas flushing, representing passive/conventional packaging in which the internal atmosphere changed only as a result of *P. pulmonarius* respiration; this treatment was defined as the control group. To simulate logistics temperature fluctuations, packaged samples were placed in an insulated foam box with ice packs and kept at 25 °C for 3 d, followed by storage at 4 °C for 2 d. This storage regime was designed to approximate short-term logistics exposure under incomplete cold-chain conditions, during which *P. pulmonarius* samples may be transported in insulated packages with ice packs and gradually warm during distribution. The subsequent 4 °C period was used to simulate short-term refrigerated storage at the retail or consumer stage. This model therefore focused on temperature fluctuation rather than mechanical vibration, stacking pressure, or other transportation-related factors. Samples were collected daily for quality, ROS metabolism, and membrane lipid metabolism analyses.

### 2.2. Optimization of Modified Atmosphere Packaging Conditions

Single-factor experiments were conducted to evaluate the effects of initial in-package O_2_ concentration, initial in-package CO_2_ concentration, and packaging film on the postharvest quality of *P. pulmonarius*. The listed O_2_ and CO_2_ concentrations refer to the initial gas composition after gas flushing, rather than constant concentrations throughout storage, because the internal atmosphere may change during storage as a result of *P. pulmonarius* respiration and film permeability.

In the O_2_ concentration experiment, five initial O_2_ levels were tested: 0%, 5%, 10%, 15%, and 20%, with 10% CO_2_ and N_2_ as the balance gas. In the CO_2_ concentration experiment, five initial CO_2_ levels were tested: 0%, 10%, 20%, 30%, and 40%, with 5% O_2_ and N_2_ as the balance gas. In the packaging film experiment, four films, namely ethylene-vinyl alcohol copolymer (EVOH), PET/PE, PA/PE, and PE films, were tested under an initial atmosphere of 5% O_2_ and 10% CO_2_.

Based on the single-factor results, an L_9_(3^3^) orthogonal design was used to further optimize MAP conditions (Table 1). Three factors were investigated: initial O_2_ concentration (A), initial CO_2_ concentration (B), and packaging film (C), with three levels for each factor. The optimal MAP condition was determined based on the comprehensive evaluation of sensory score, browning index (BI), and malondialdehyde (MDA)content.

### 2.3. Quality Evaluation

#### 2.3.1. Sensory Evaluation

Sensory evaluation was performed according to previously reported methods for mushroom quality assessment, with slight modifications [9]. Eight trained researchers evaluated the sensory quality of *P. pulmonarius* based on color, odor, morphology, texture, and decay degree. The total sensory score was 100 points. Each sample was evaluated independently, and the average score was used for analysis. The detailed scoring criteria are shown in Table 2.

#### 2.3.2. Weight Loss

Weight loss during storage was determined according to the method described by Zhu et al. [15]. The initial weight and the weight after storage were recorded, and weight loss was calculated using the following equation:

(1) $$Weight\;loss\left(\%\right)=\;\frac{W-W_{1}}{W}\times 100\%$$

where W represents the initial weight of the sample, and W_1_ represents the weight of the sample after storage.

#### 2.3.3. Browning Index

The browning index (BI) was determined according to the method described by Fan et al. [16]. The color parameters L*, a*, and b* were measured using a colorimeter (NS810, Shenzhen Threenh Technology Co., Ltd., Shenzhen, China). BI was calculated using the following equations:

(2) $$BI=(\frac{a^{*}+{1.75\times L}^{*}}{{5.645\times L}^{*}+a^{*}-{3.012\times b}^{*}}-0.31)÷0.172\times 100$$

where L*, a*, and b* represent the measured color parameters.

#### 2.3.4. Determination of Total Phenolic and Flavonoid Contents

Total phenolic and flavonoid contents were determined colorimetrically according to Yuan et al. [17], with slight modifications. Briefly, 0.5 g of *P. pulmonarius* tissue was extracted with 5 mL of pre-cooled 1% hydrochloric acid–methanol solution and centrifuged at 8000 r/min at 4 °C. The supernatant was used for analysis.

Total phenolic content was determined using the Folin–Ciocalteu method with gallic acid as the standard, and absorbance was measured at 765 nm. Flavonoid content was determined using the sodium nitrite–aluminum nitrate colorimetric method with rutin as the standard, and absorbance was measured at 510 nm. The results were expressed as mg gallic acid equivalents/g fresh weight and mg rutin equivalents/g fresh weight, respectively.

### 2.4. Determination of Reactive Oxygen Species Metabolism-Related Indicators

#### 2.4.1. Determination of Superoxide Anion Production Rate

The superoxide anion (O_2_^.^^−^) production rate was determined using a commercial superoxide anion assay kit (Fujian Herui Biotechnology Co., Ltd., Fuzhou, China) according to the manufacturer’s instructions. The results were expressed as nmol/(g·min).

#### 2.4.2. Determination of Malondialdehyde Content

Malondialdehyde (MDA) content was determined using the thiobarbituric acid method described by Chen et al. [18]. Briefly, 0.1 g of *P. pulmonarius* sample was homogenized with 1 mL of trichloroacetic acid solution and centrifuged at 8000 r/min at 4 °C for 10 min. Then, 0.1 mL of the supernatant was mixed with 0.3 mL of thiobarbituric acid solution and heated in a water bath at 95 °C for 30 min. After rapid cooling and centrifugation, the absorbance of the supernatant was measured at 532 and 600 nm. The MDA content was expressed as nmol/g.

#### 2.4.3. Determination of SOD, CAT, and APX Activities

The crude enzyme extract was prepared by homogenizing 1 g of *P. pulmonarius* tissue with 50 mmol/L phosphate-buffered saline (PBS, pH 7.0), followed by centrifugation at 10,000 r/min at 4 °C for 15 min. The supernatant was used for enzyme activity assays. SOD, CAT, and APX activities were determined according to previously reported methods, with slight modifications [11,19,20,21]. SOD activity was measured based on the inhibition of nitroblue tetrazolium reduction at 560 nm. CAT activity was determined by monitoring the decomposition of H_2_O_2_ at 240 nm. APX activity was determined by monitoring the oxidation of ascorbic acid at 290 nm. Enzyme activities were expressed as U/mg protein.

#### 2.4.4. Determination of AsA and GSH Contents

AsA and GSH contents were determined using a trichloroacetic acid extract according to previously reported methods, with slight modifications [22]. Briefly, 1 g of sample was homogenized with 5 mL of pre-cooled 5% trichloroacetic acid and centrifuged at 12,000 r/min at 4 °C for 10 min. The supernatant was collected for analysis. AsA content was determined colorimetrically at 534 nm using ascorbic acid as the standard, while GSH content was determined at 412 nm using reduced glutathione as the standard. The results were expressed as mg/100 g and μmol/g, respectively.

#### 2.4.5. Determination of DPPH Radical Scavenging Capacity and Reducing Power

DPPH radical scavenging capacity and reducing power were determined using an ethanol extract. Briefly, 1 g of *P. pulmonarius* tissue was homogenized with 5 mL of 80% ethanol and centrifuged at 8000 r/min for 10 min. DPPH radical scavenging capacity was determined by mixing the extract with DPPH ethanol solution and measuring absorbance at 517 nm [22]. Reducing power was determined according to Gu et al. [23] by measuring absorbance at 700 nm after reaction with potassium ferricyanide and ferric chloride. DPPH radical scavenging capacity was expressed as a percentage and calculated as follows:

(3) $$DPPH\;radical\;scavenging\;capacity\left(\%\right)=\;\frac{A-B}{A}\times 100\%$$

where A represents the absorbance of the blank control, and B represents the absorbance of the sample.

### 2.5. Determination of Membrane Lipid Metabolism-Related Indicators

#### 2.5.1. Determination of Membrane Permeability

Membrane permeability was determined according to the method described by Gao et al. [24]. Briefly, *P. pulmonarius* samples were cut into 1 cm × 1 cm × 1 cm cubes, and 5 g of tissue was weighed. The tissue cubes were washed three times with deionized water, drained, and then immersed in 50 mL of deionized water. After incubation at 30 °C for 1 h, the initial electrical conductivity was measured and recorded as (P_0). The samples were then boiled in a water bath for 15 min and cooled to room temperature, after which the final electrical conductivity was measured and recorded as (P_1). Membrane permeability was calculated using the following equation:

(4) $$Membrane\;permeability\left(\%\right)=\;\frac{P_{0}}{P_{1}}\times 100\%$$

where P_0_ represents the electrical conductivity before boiling, and P_1_ represents the electrical conductivity after boiling and cooling.

#### 2.5.2. Determination of PLD, Lipase, and LOX Activities

The activities of membrane lipid degradation-related enzymes were determined spectrophotometrically according to previously reported methods, with slight modifications. Lipase activity was determined using 1-naphthyl acetate as the substrate, and absorbance was measured at 520 nm [25]. LOX activity was determined using sodium linoleate as the substrate, and absorbance was measured at 234 nm [26]. PLD activity was determined using lecithin as the substrate, followed by color development with Reinecke salt, and absorbance was measured at 520 nm [27]. All enzyme activities were expressed as U/mg protein.

#### 2.5.3. Determination of Phospholipid Components

Phospholipid components were extracted and analyzed by high-performance liquid chromatography (HPLC) according to previously reported methods, with slight modifications [28]. Briefly, 10 g of *P. pulmonarius* tissue was extracted with chloroform–methanol solution, followed by centrifugation, nitrogen drying, re-dissolution, and filtration through a 0.22 μm membrane. Phosphatidylcholine (PC), phosphatidylinositol (PI), and phosphatidic acid (PA) were identified and quantified using corresponding standards. HPLC analysis was performed on an Intersil SIL 100A column (4.6 mm × 250 mm) at 30 °C, with a flow rate of 0.8 mL/min, detection wavelength of 254 nm, and injection volume of 10 μL. The contents of PC, PI, and PA were expressed as mg/g.

#### 2.5.4. Determination of Membrane Fatty Acid Composition

Membrane fatty acid composition was determined according to the method described by Niu et al. [12], with slight modifications. The *P. pulmonarius* samples were first heated at 105 °C for 30 min to inactivate enzymes and then ground into powder. Subsequently, 0.5 g of sample powder was mixed with 1.5 mL of 0.4 mol/L KOH–methanol solution and shaken for 4 h. Then, 1.5 mL of benzene–petroleum ether solution was added, followed by vortex mixing and standing for 30 min. After the addition of 1.5 mL of distilled water, the mixture was vortexed again and allowed to stand for phase separation. The upper phase was collected, filtered through a 0.22 μm membrane, and used for gas chromatography (GC) analysis.

Fatty acid components were identified according to the retention times of standard fatty acids. The peak areas of saturated fatty acids (SFAs) and unsaturated fatty acids (USFAs) were integrated and normalized. GC analysis was performed using a GC-2010 Plus system equipped with a BD-23 column (Shimadzu, Kyoto, Japan). The injector temperature was 270 °C, the flame ionization detector temperature was 280 °C, the initial column temperature was 50 °C, and the maximum column temperature was 245 °C. The flow rates of hydrogen, air, and nitrogen were 30, 400, and 40 mL/min, respectively.

The unsaturated-to-saturated fatty acid ratio (U/S) and the index of unsaturated fatty acids (IUFA) were calculated using the following equations:

(5) $$U/S=\;\frac{USFAs}{SFAs}$$

(6) $$IUFA=[\sum_{i}^{n}\left(S_{i}\times T_{i}\right)]\times 100\%$$

where S_i_ represents the relative content of each unsaturated fatty acid, and T_i_ represents the number of double bonds in the corresponding unsaturated fatty acid.

### 2.6. Data Processing and Statistical Analysis

All experiments were conducted using three biological replicates, with each replicate corresponding to an independently packaged sample. The results are expressed as mean ± standard deviation (SD). Statistical analyses were performed using jamovi software (Version 2.6). Differences between MAP and control samples at the same sampling time were analyzed using an independent-samples *t*-test. For optimization experiments, one-way analysis of variance (ANOVA) was used to evaluate differences among treatments. Differences were considered significant at *p* < 0.05 and highly significant at *p* < 0.01.

## 3. Results

### 3.1. Optimization and Validation of Modified Atmosphere Packaging Conditions Under Simulated Logistics

#### 3.1.1. Temperature Changes During Simulated Logistics

**Figure 1:**
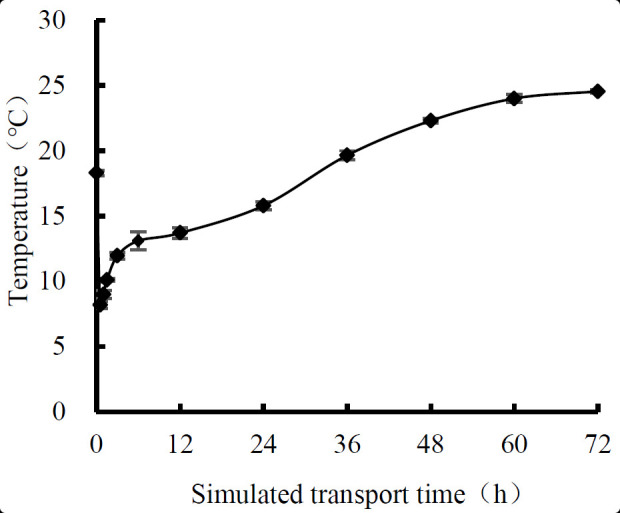
Changes in the temperature inside the foam box during simulated logistics.

The temperature profile inside the foam box during simulated logistics is shown in Figure 1. The internal temperature initially decreased from 18.30 °C to 8.20 °C within 0.5 h because of the cooling effect of the ice packs. Thereafter, the temperature gradually increased as the cooling capacity declined, reaching 15.80 °C at 24 h and 24.55 °C at 72 h, which was close to the external simulated logistics temperature of 25 °C. These results indicate that the simulated logistics system reproduced a typical short-term temperature fluctuation process, with an early cooling stage followed by gradual warming.

#### 3.1.2. Single-Factor Experiments for Optimizing Modified Atmosphere Packaging Conditions

**Figure 2:**
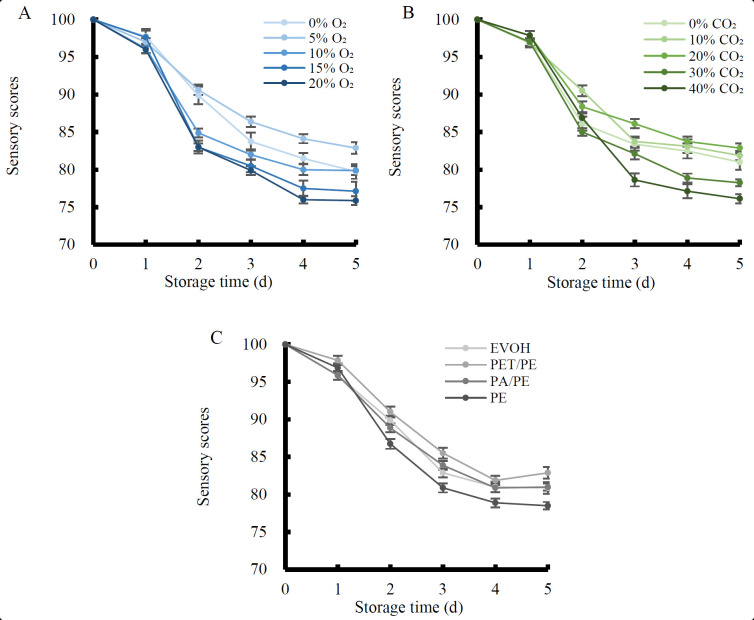
Effects of different O_2_ concentrations (**A**), CO_2_ concentrations (**B**), and sealing films (**C**) on the sensory score of *P. pulmonarius*.

**Figure 3:**
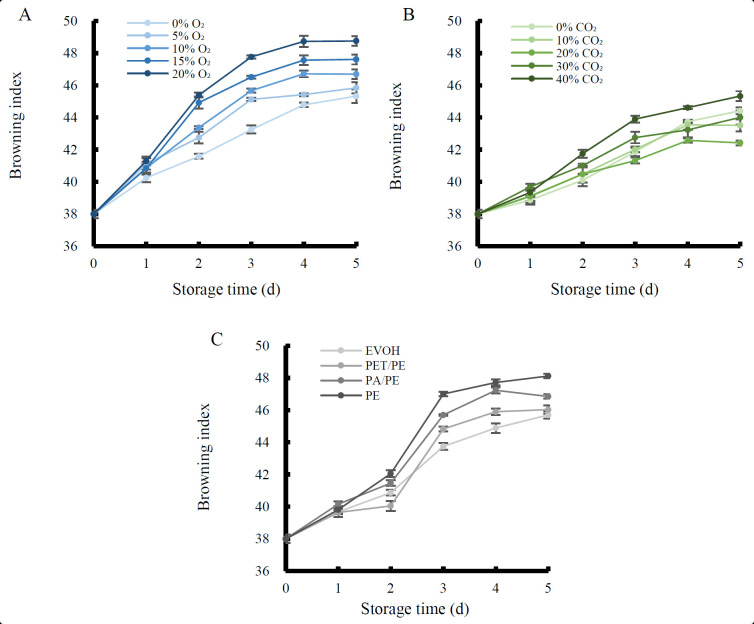
Effects of different O_2_ concentrations (**A**), CO_2_ concentrations (**B**), and sealing films (**C**) on the browning index of *P. pulmonarius*.

**Figure 4:**
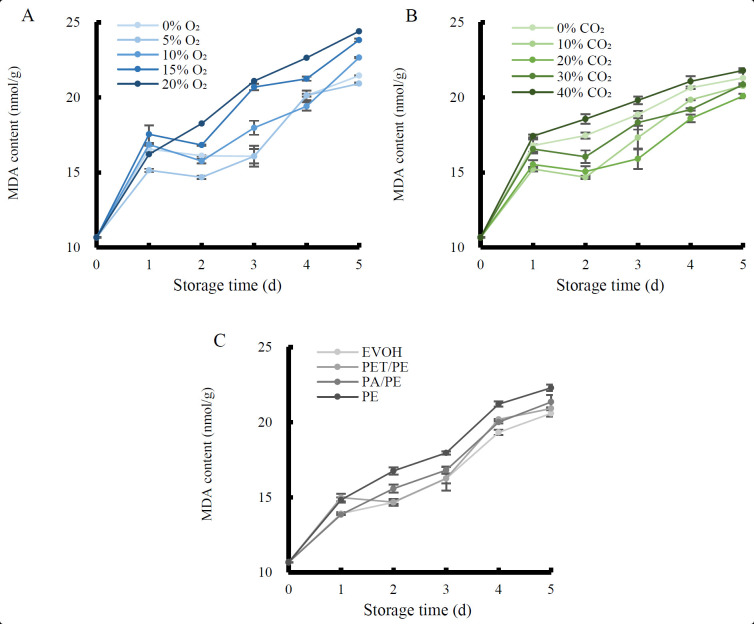
Effects of different O_2_ concentrations (**A**), CO_2_ concentrations (**B**), and sealing films (**C**) on malondialdehyde content in *P. pulmonarius*.

The effects of O_2_ concentration, CO_2_ concentration, and packaging film on the sensory score, browning index (BI), and MDA content of *P. pulmonarius* are shown in Figure 2, Figure 3 and Figure 4. During storage, sensory scores decreased, whereas BI and MDA content increased in all treatments, indicating progressive quality deterioration under simulated logistics. However, the extent of deterioration varied markedly among treatments.

For O_2_ concentration, 5% O_2_ maintained the highest sensory score and the lowest MDA content at the end of storage, while higher O_2_ concentrations, especially 15% and 20%, accelerated browning and MDA accumulation. The 0% O_2_ treatment showed relatively low BI but caused an obvious alcoholic odor, suggesting that complete oxygen depletion was not suitable for maintaining overall sensory quality. Therefore, 5% O_2_ was considered the most appropriate O_2_ concentration among the tested levels.

For CO_2_ concentration, 20% CO_2_ showed the best overall preservation performance, maintaining the highest sensory score and the lowest BI and MDA content at 5 d. In contrast, excessive CO_2_, particularly 40%, reduced sensory quality and increased MDA accumulation, probably because high CO_2_ levels induced anaerobic metabolism and physiological stress. These results indicate that moderate CO_2_ enrichment was beneficial for delaying quality deterioration.

Packaging film also affected storage quality. PET/PE film showed the highest sensory score, whereas EVOH film resulted in relatively low MDA content. PE film showed poorer performance, with lower sensory scores and higher BI and MDA values. Considering sensory quality and oxidative damage together, EVOH, PET/PE, and PA/PE films were selected for further orthogonal optimization.

#### 3.1.3. Orthogonal Optimization of Modified Atmosphere Packaging Conditions

Based on the single-factor experiments, an L9(3^3^) orthogonal design was used to optimize the MAP conditions. Three factors were investigated: O_2_ concentration (A), CO_2_ concentration (B), and packaging film material (C), with sensory score, BI, and MDA content as evaluation indicators. The orthogonal experimental results are shown in Table 3.

**Figure 5:**
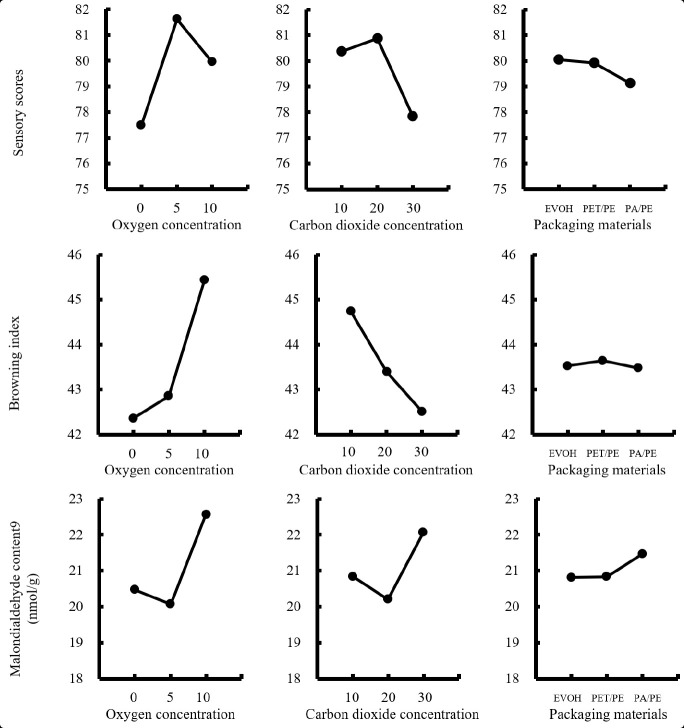
Relationships between the experimental factors and evaluation indicators.

Range analysis showed that the influence of the three factors on sensory score, BI, and MDA content followed the same order: O_2_ concentration (A) > CO_2_ concentration (B) > packaging film material (C) (Table 4 and Figure 5). Although the lowest BI was obtained under 0% O_2_, 30% CO_2_, and PA/PE film, this condition was not selected because the 0% O_2_ treatment caused off-odor formation in the single-factor experiment, indicating a potential risk of anaerobic metabolism. Therefore, sensory score and MDA content were used as primary decision indicators, while BI was considered as a supporting indicator. Sensory score was prioritized because it directly reflects consumer acceptability and overall commercial quality, whereas MDA content was used as an indicator of oxidative membrane damage. Based on this rationale and the subsequent validation test, 5% O_2_, 20% CO_2_, and EVOH film were selected as the optimal MAP condition.

The ANOVA results further confirmed that O_2_ and CO_2_ concentrations were the dominant factors affecting storage quality (Table 5). Factors A and B significantly affected sensory score, BI, and MDA content, whereas the effect of packaging film was not significant. These results were consistent with the range analysis and indicated that gas composition played a more important role than film type in determining MAP performance.

#### 3.1.4. Validation Test

Two candidate combinations were selected for validation: combination 1, consisting of 5% O_2_, 20% CO_2_, and EVOH film; and combination 2, consisting of 0% O_2_, 30% CO_2_, and PA/PE film. As shown in Table 6, combination 1 resulted in significantly higher sensory score, hardness, soluble protein content, and total sugar content, together with lower MDA content and membrane permeability. Although combination 2 showed a slightly lower BI, its poorer sensory quality and higher membrane damage indicators suggested inferior overall preservation performance. Therefore, 5% O_2_, 20% CO_2_, and EVOH film were selected as the optimal MAP conditions for *P. pulmonarius* under simulated logistics.

### 3.2. Effects of MAP on the Quality and Physicochemical Properties of P. pulmonarius Under Simulated Logistics

#### 3.2.1. Sensory Score and Appearance

**Figure 6:**
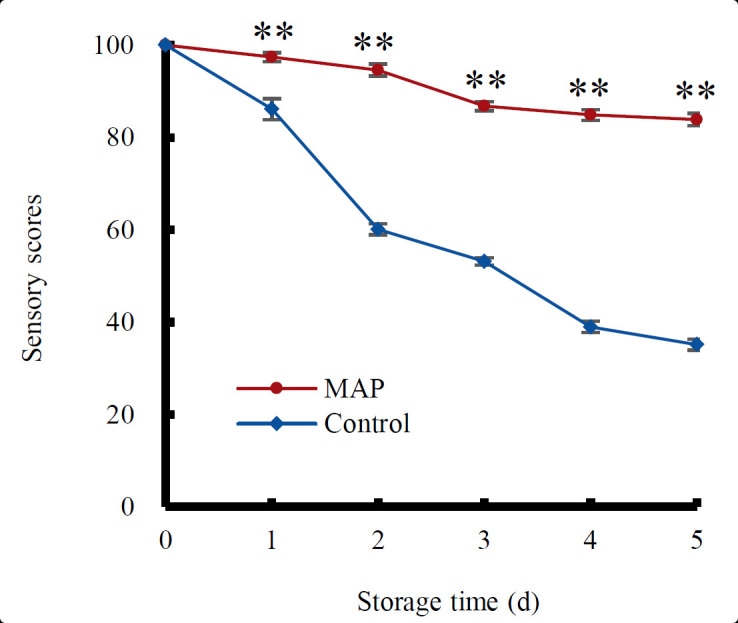
Effect of different treatments on the sensory score of *P. pulmonarius*. Asterisks (**) indicate significant differences (*p* < 0.01) between the MAP group and the Control group.

**Figure 7:**
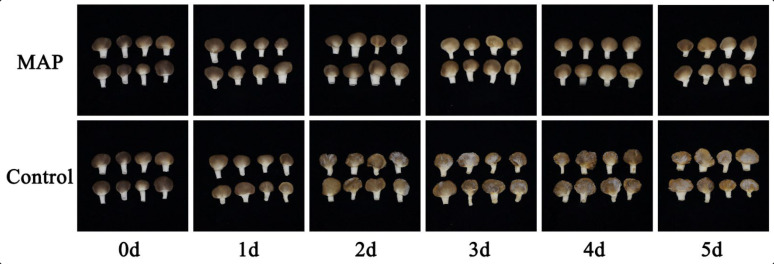
Effect of different treatments on the appearance of *P. pulmonarius*.

As shown in Figure 6 and Figure 7, sensory quality decreased in both groups during storage, but the decline was much slower in the MAP group. The control samples showed rapid quality deterioration from 2 d onward, accompanied by mold growth, browning, and spoilage odor. By contrast, MAP-treated samples maintained good appearance quality throughout storage, with only slight browning at 5 d. At the end of storage, the sensory score of the MAP group remained 83.88, whereas that of the control group decreased to 35.13 (*p* < 0.01). These results indicate that MAP effectively maintained the visual and sensory quality of *P. pulmonarius* under simulated logistics.

#### 3.2.2. Weight Loss and Browning Index

**Figure 8:**
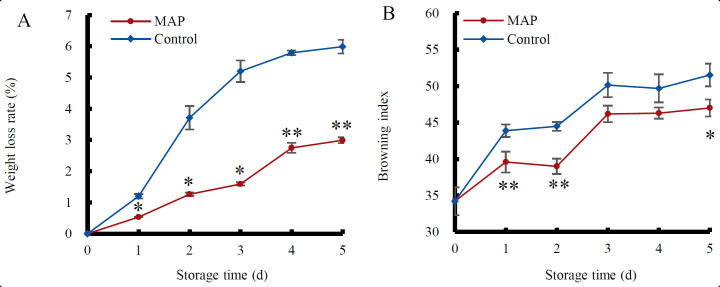
Effect of different treatments on the weight loss rate (**A**) and browning index (**B**) of *P. pulmonarius*. Asterisks (* and **) indicate significant differences (*p* < 0.05 and *p* < 0.01, respectively) between the MAP group and the Control group.

As shown in Figure 8A, weight loss increased in both groups during storage, but the increase was significantly lower in the MAP group. At 5 d, the weight loss rate of MAP-treated samples was 2.99%, approximately half that of the control group. This indicates that MAP effectively reduced water loss during simulated logistics and subsequent storage.

The BI of *P. pulmonarius* increased during storage, indicating progressive browning (Figure 8B). However, the MAP group consistently showed lower BI values than the control group, especially during the early simulated logistics stage. At 5 d, the BI values of the MAP and control groups were 47.01 and 51.53, respectively. These results suggest that MAP delayed browning and helped maintain the color quality of *P. pulmonarius*.

#### 3.2.3. Total Phenolic and Flavonoid Contents

**Figure 9:**
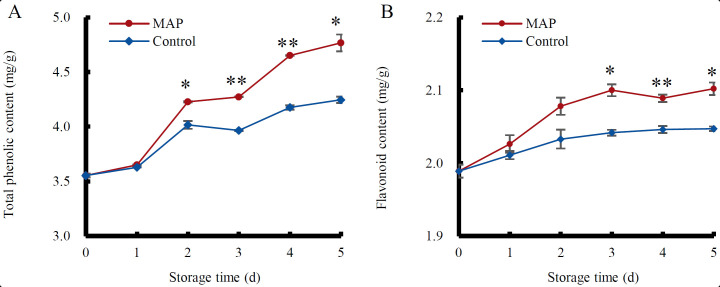
Effects of different treatments on total phenolic content (**A**) and flavonoid content (**B**) of *P. pulmonarius*. Asterisks (* and **) indicate significant differences (*p* < 0.05 and *p* < 0.01, respectively) between the MAP group and the Control group.

The total phenolic and flavonoid contents of *P. pulmonarius* generally increased during storage (Figure 9). Compared with the control, MAP treatment maintained higher total phenolic content at 1 and 3–5 d, with the highest value of 4.77 mg/g at 5 d. Flavonoid content showed a similar pattern, and the MAP group was significantly higher than the control at 3–5 d. These results indicate that MAP helped preserve antioxidant-related compounds in *P. pulmonarius* during simulated logistics and storage.

### 3.3. Effects of MAP on Reactive Oxygen Species Metabolism in P. pulmonarius Under Simulated Logistics

#### 3.3.1. MDA Content and Superoxide Anion Production Rate

**Figure 10:**
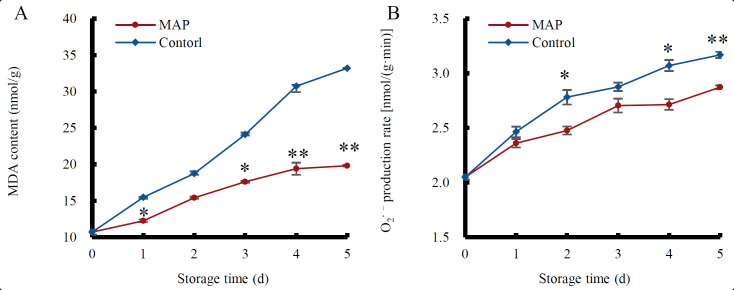
Effects of different treatments on malondialdehyde content (**A**) and superoxide anion production rate (**B**) in *P. pulmonarius*. Asterisks (* and **) indicate significant differences (*p* < 0.05 and *p* < 0.01, respectively) between the MAP group and the Control group.

As shown in Figure 10, both MDA content and O_2_^.^^−^ production rate increased during storage, indicating enhanced oxidative stress in *P. pulmonarius*. MAP treatment significantly inhibited the accumulation of both indicators. At 5 d, the MDA content in the MAP group was 19.814 nmol/g, accounting for only 59.65% of that in the control group. Similarly, the O_2_^.^^−^ production rate in the MAP group remained lower than that in the control group during storage and was significantly lower at 2, 4, and 5 d. These results suggest that MAP alleviated oxidative stress by suppressing ROS accumulation and membrane lipid peroxidation.

#### 3.3.2. Activities of ROS-Scavenging Enzymes and Contents of Non-Enzymatic Antioxidants

**Figure 11:**
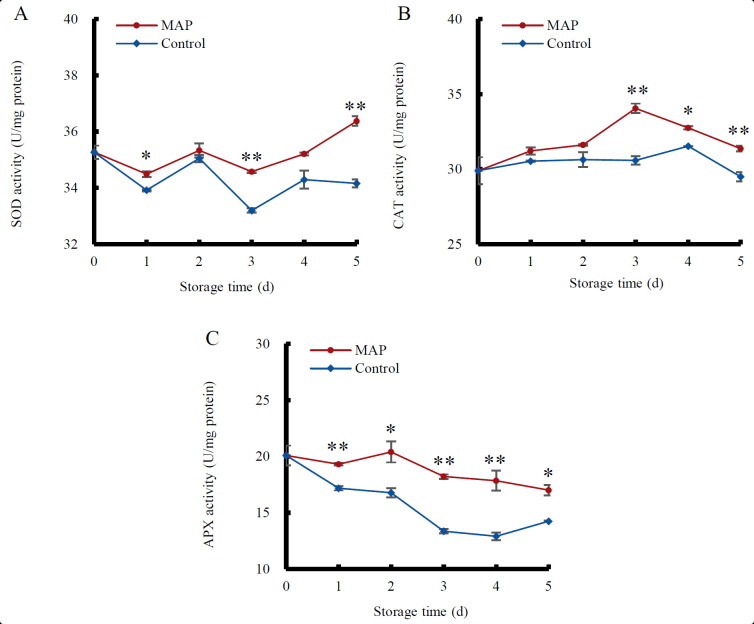
Effects of different treatments on the activities of ROS-scavenging enzymes, including SOD (**A**), CAT (**B**), and APX (**C**), in *P. pulmonarius*. Asterisks (* and **) indicate significant differences (*p* < 0.05 and *p* < 0.01, respectively) between the MAP group and the Control group.

**Figure 12:**
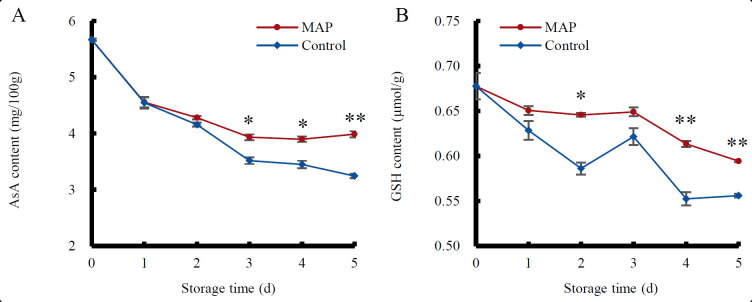
Effects of different treatments on the contents of non-enzymatic ROS-scavenging substances, including AsA (**A**) and GSH (**B**), in *P. pulmonarius*. Asterisks (* and **) indicate significant differences (*p* < 0.05 and *p* < 0.01, respectively) between the MAP group and the Control group.

MAP treatment enhanced the ROS-scavenging capacity of *P. pulmonarius* during storage (Figure 11 and Figure 12). SOD activity fluctuated but remained higher in the MAP group, particularly at 3 and 5 d. CAT activity increased initially and then decreased, with MAP-treated samples showing significantly higher activity from 3 to 5 d. APX activity declined during storage, but the MAP group maintained higher APX activity than the control throughout most of the storage period.

The non-enzymatic antioxidants AsA and GSH decreased during storage, but their depletion was delayed by MAP treatment. At 5 d, AsA and GSH contents in the MAP group were significantly higher than those in the control. These results indicate that MAP maintained ROS homeostasis by enhancing enzymatic antioxidant activities and preserving non-enzymatic antioxidants.

#### 3.3.3. DPPH Radical Scavenging Capacity and Reducing Power

**Figure 13:**
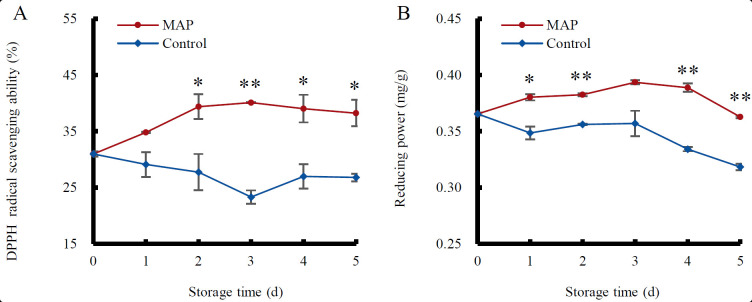
Effects of different treatments on DPPH radical scavenging capacity (**A**) and reducing power (**B**) of *P pulmonarius*. Asterisks (* and **) indicate significant differences (*p* < 0.05 and *p* < 0.01, respectively) between the MAP group and the Control group.

As shown in Figure 13, MAP-treated samples maintained higher antioxidant capacity than the control during storage. DPPH radical scavenging capacity increased in the MAP group during the early storage stage and remained significantly higher than that in the control from 2 to 5 d. Reducing power showed a similar trend, with higher values observed in the MAP group, especially at 2, 4, and 5 d. These results further indicate that MAP enhanced the antioxidant capacity of *P. pulmonarius* under simulated logistics.

### 3.4. Effects of MAP on Membrane Lipid Metabolism in P. pulmonarius Under Simulated Logistics

#### 3.4.1. Membrane Permeability

**Figure 14:**
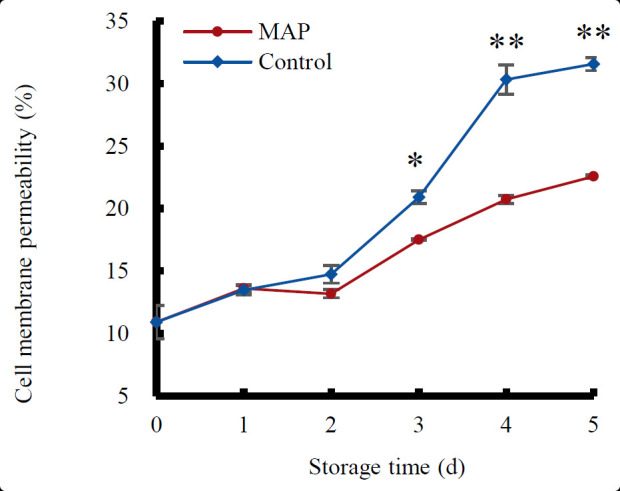
Effects of different treatments on membrane permeability of *P pulmonarius*. Asterisks (* and **) indicate significant differences (*p* < 0.05 and *p* < 0.01, respectively) between the MAP group and the Control group.

Membrane permeability increased continuously during storage, indicating progressive membrane damage (Figure 14). The increase was much faster in the control group, especially from 2 to 4 d. At 5 d, membrane permeability reached 31.57% in the control group, whereas the MAP group remained at 22.57%. The significantly lower membrane permeability in MAP-treated samples indicates that MAP effectively maintained membrane integrity during simulated logistics.

#### 3.4.2. Activities of Membrane Lipid Degradation-Related Enzymes

**Figure 15:**
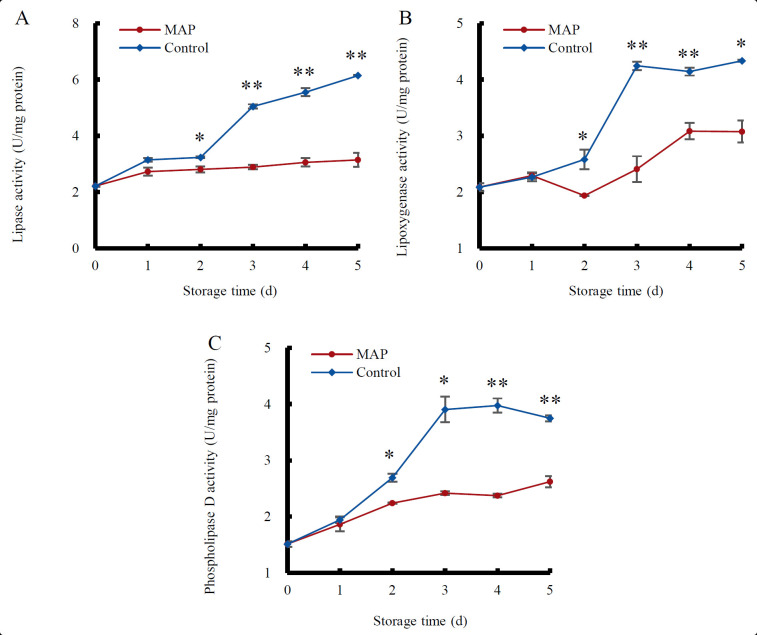
Effects of different treatments on membrane lipid degradation-related enzymes, including lipase (**A**), LOX (**B**), and PLD (**C**), in *P. pulmonarius*. Asterisks (* and **) indicate significant differences (*p* < 0.05 and *p* < 0.01, respectively) between the MAP group and the Control group.

As shown in Figure 15, the activities of lipase, LOX, and PLD increased during storage, indicating enhanced membrane lipid degradation. However, MAP treatment significantly suppressed the activities of all three enzymes, particularly during the later storage period. At 5 d, lipase, LOX, and PLD activities in the MAP group were markedly lower than those in the control group. These results indicate that MAP delayed membrane lipid degradation by inhibiting lipid-degrading enzymes.

#### 3.4.3. Contents of Phospholipid Components

**Figure 16:**
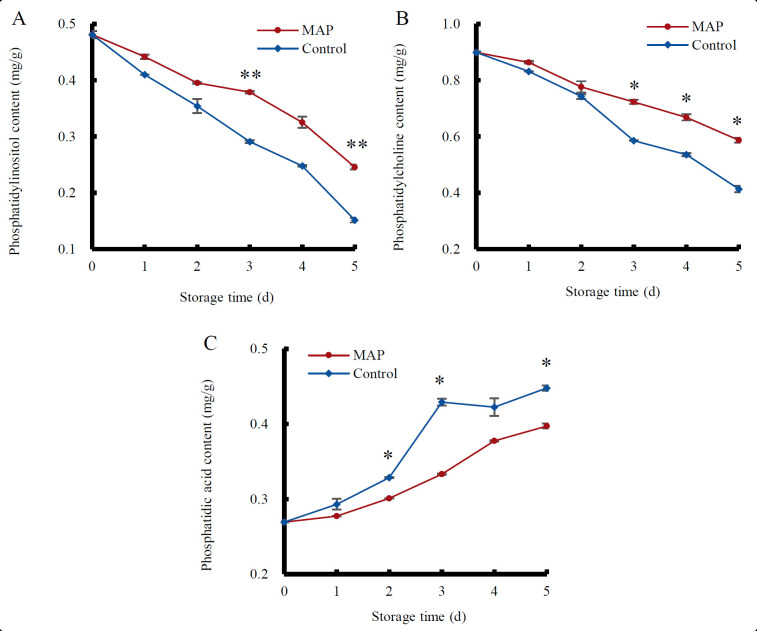
Effects of different treatments on phospholipid components, including PI (**A**), PC (**B**), and PA (**C**), in *P. pulmonarius*. Asterisks (* and **) indicate significant differences (*p* < 0.05 and *p* < 0.01, respectively) between the MAP group and the Control group.

The contents of PI and PC decreased during storage, whereas PA content increased (Figure 16). Compared with the control, MAP treatment maintained higher PI and PC contents and inhibited PA accumulation, especially during the later storage stage. At 5 d, PI and PC contents in the MAP group were 1.62 and 1.44 times those in the control group, respectively, while PA content remained lower. These results indicate that MAP delayed phospholipid degradation and helped maintain membrane lipid stability.

#### 3.4.4. Membrane Fatty Acid Composition

Fresh *P. pulmonarius* membrane lipids were dominated by unsaturated fatty acids (USFAs), which accounted for 89.44% of total fatty acids at 0 d (Table 7). Linoleic acid was the predominant fatty acid, accounting for 78.44% of total fatty acids.

**Figure 17:**
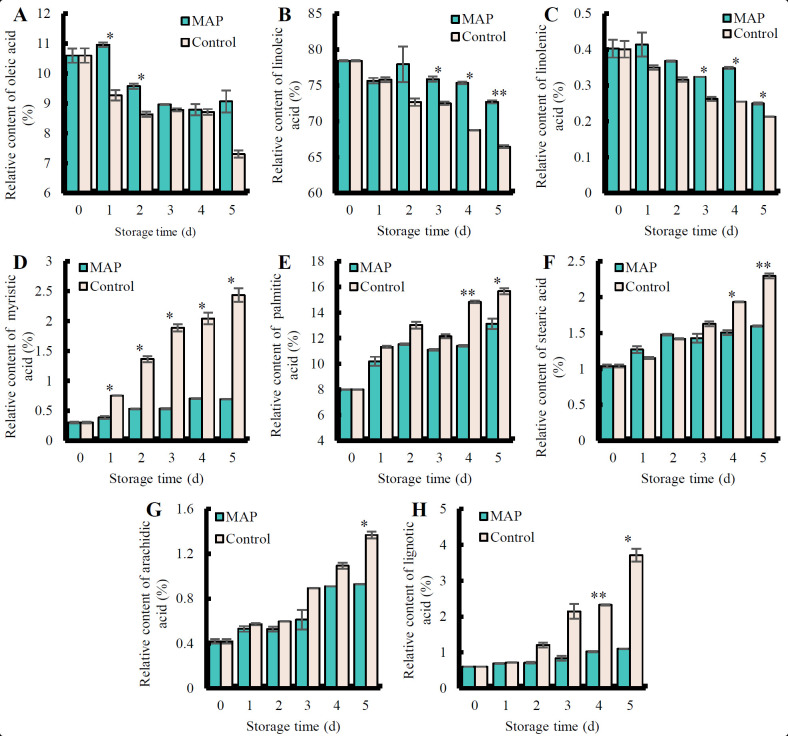
Effects of different treatments on membrane fatty acid components, including Oleic acid (**A**), Linoleic acid (**B**), Linolenic acid (**C**), Myristic acid (**D**), Palmitic acid (**E**), Stearic acid (**F**), Arachidic acid (**G**), and Lignoceric acid (**H**), in *P. pulmonarius*. Asterisks (* and **) indicate significant differences (*p* < 0.05 and *p* < 0.01, respectively) between the MAP group and the Control group.

During storage, USFAs, including oleic acid, linoleic acid, and linolenic acid, decreased, whereas saturated fatty acids (SFAs), including myristic, palmitic, stearic, arachidic, and lignoceric acids, increased (Figure 17). MAP treatment delayed the decline in USFAs and suppressed the accumulation of SFAs. In particular, linoleic acid and linolenic acid contents were significantly higher in the MAP group during the later storage period, while the contents of major SFAs were lower than those in the control.

**Figure 18:**
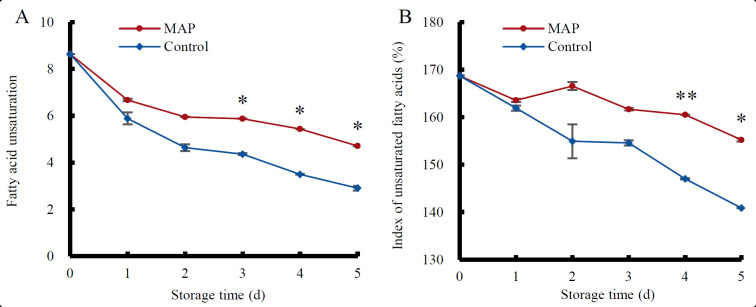
Effects of different treatments on membrane fatty acid unsaturation (**A**) and index of unsaturated fatty acids (**B**) of *P. pulmonarius*. Asterisks (* and **) indicate significant differences (*p* < 0.05 and *p* < 0.01, respectively) between the MAP group and the Control group.

Consistent with these changes, both U/S and IUFA decreased during storage, but MAP-treated samples maintained significantly higher values than the control at the later storage stages (Figure 18). These results indicate that MAP helped preserve membrane fatty acid unsaturation and membrane lipid stability during simulated logistics.

## 4. Discussion

### 4.1. Optimization of MAP Conditions for P. pulmonarius Under Simulated Logistics

In this study, a simulated logistics system was established to reproduce temperature fluctuations during short-term transportation. The temperature inside the foam box initially decreased because of the cooling effect of ice packs, but then gradually increased and approached the external temperature of 25 °C after 72 h. Similar laboratory-scale logistics models have been used to evaluate packaging microenvironments and quality changes in fresh horticultural products during transportation [7]. These results indicate that simulated logistics can provide a practical approach for assessing postharvest preservation strategies under fluctuating temperature conditions.

The single-factor experiments showed that the quality responses of *P. pulmonarius* differed among packages flushed with different initial O_2_ and CO_2_ compositions. Treatments with excessive initial CO_2_ or higher initial O_2_ levels generally showed poorer sensory quality and/or higher oxidative damage indicators. According to previous studies, excessively low O_2_ or high CO_2_ atmospheres may induce anaerobic metabolism, leading to ethanol and acetaldehyde accumulation and undesirable fermented odors [29]. Excessive CO_2_ may also cause physiological stress and cellular injury, whereas high O_2_ availability may accelerate respiratory metabolism, ROS production, and membrane lipid peroxidation, thereby promoting browning and oxidative damage. Therefore, an appropriate initial balance between O_2_ reduction and CO_2_ enrichment is important for MAP preservation. However, because dynamic headspace O_2_ and CO_2_ concentrations were not monitored during storage, these results should be interpreted as responses to the initial MAP settings rather than to constant in-package gas concentrations throughout the 5-day storage period. Previous studies on button mushrooms also showed that relatively low O_2_ and elevated CO_2_ atmospheres were effective in maintaining appearance, inhibiting browning-related enzyme activity, and prolonging shelf life [8].

Packaging film also influenced the preservation effect, probably because different films differ in gas permeability and gas selectivity. In the present study, EVOH, PET/PE, and PA/PE films showed better preservation performance than PE film during the optimization experiments. Similar findings have been reported for *Pleurotus nebrodensis*, where PE films with different thicknesses altered the package atmosphere and affected mushroom quality [9]. Based on the single-factor, orthogonal, and validation experiments, the optimal MAP condition was 5% O_2_, 20% CO_2_, and EVOH film. Under this condition, *P. pulmonarius* maintained higher sensory quality, lower weight loss, lower MDA content, and lower membrane permeability at the end of simulated logistics and storage, indicating that this gas–film combination effectively delayed postharvest deterioration. However, because dynamic changes in O_2_ and CO_2_ concentrations were not monitored during storage, the physiological suitability of the high-barrier EVOH-based MAP system should be interpreted with caution. This limitation is particularly relevant for the EVOH-based MAP system because EVOH film has a very low OTR. Although this high-barrier property may help maintain the initial modified atmosphere during short-term storage, prolonged storage could increase the risk of excessive O_2_ depletion after the initial oxygen is consumed by *P. pulmonarius* respiration. Under such conditions, anaerobic metabolism, off-odor formation, and physiological injury may occur. Therefore, the suitability of the optimized EVOH-based MAP treatment should be limited to the 5-day simulated logistics and cold storage period tested in this study. For longer commercial storage or more variable distribution conditions, dynamic headspace gas monitoring and further optimization of film permeability are necessary.

### 4.2. Effects of MAP on the Quality and Physicochemical Characteristics of P. pulmonarius Under Simulated Logistics

Fresh mushrooms are highly perishable because of their high moisture content, active metabolism, and fragile tissue structure. Water loss, browning, texture deterioration, microbial spoilage, and nutrient depletion are major causes of postharvest quality deterioration in mushrooms [3,4]. Storage temperature and packaging conditions further influence quality by regulating respiration, microbial growth, and physiological senescence [5,6]. In this study, sensory scores decreased and weight loss increased during storage in both groups, but MAP-treated samples consistently showed higher sensory scores and lower weight loss than the control. This suggests that MAP reduced water loss and slowed quality deterioration during simulated logistics. In addition to water loss, browning, and tissue softening, spoilage-related symptoms such as visible mold growth, off-odor, and decay degree also contributed to the decline in sensory quality.

Color deterioration is one of the most visible indicators of mushroom senescence. In the present study, the browning index increased throughout storage, indicating progressive browning of *P. pulmonarius*. However, MAP treatment significantly slowed the increase in browning index compared with the control. This may be attributed to the reduced O_2_ availability inside the package, which limited oxidative reactions and suppressed browning-related physiological changes. Previous studies have also shown that storage temperature and packaging conditions strongly affect color, microbial quality, and overall postharvest quality of mushrooms [5,6].

Total phenolics and flavonoids are important antioxidant-related compounds that contribute to stress resistance and browning regulation. In this study, MAP-treated samples maintained higher total phenolic and flavonoid contents than the control during storage. The accumulation or retention of phenolic compounds may be related to stress-induced metabolic regulation and reduced oxidative consumption. Similar mechanisms have been reported in harvested fruit, where preservation treatments delayed browning and senescence by regulating ROS metabolism and alleviating oxidative damage [30]. Therefore, MAP maintained the postharvest quality of *P. pulmonarius* by reducing water loss, delaying browning, and preserving antioxidant-related functional compounds.

### 4.3. Effects of ROS Metabolism on the Quality of MAP-Treated P. pulmonarius Under Simulated Logistics

ROS are highly reactive oxygen-containing molecules that play dual roles in plant and fungal tissues. At low levels, ROS can function as signaling molecules, but excessive ROS accumulation causes oxidative damage to lipids, proteins, and nucleic acids [31]. During postharvest storage, respiratory metabolism and environmental stress promote ROS accumulation, which can trigger membrane lipid peroxidation and accelerate senescence. MDA is a typical product of lipid peroxidation and is commonly used as an indicator of oxidative membrane damage.

In this study, both O_2_^.^^−^ production rate and MDA content increased during storage, indicating enhanced oxidative stress in *P. pulmonarius*. However, MAP treatment significantly inhibited the accumulation of O_2_^.^^−^ and MDA. Correlation analysis further showed that O_2_^.^^−^ production rate and MDA content were negatively correlated with storage quality, suggesting that oxidative damage was closely associated with quality deterioration. Similar results were reported in apricot fruit, where the combined treatment of intense pulsed light and MAP inhibited O_2_^.^^−^, H_2_O_2_, and MDA accumulation and delayed browning [32]. These findings indicate that MAP delayed oxidative deterioration of *P. pulmonarius* by suppressing ROS accumulation and subsequent lipid peroxidation.

The antioxidant defense system plays a key role in maintaining ROS homeostasis. SOD converts O_2_^.^^−^ into H_2_O_2_, while CAT and APX further decompose H_2_O_2_. In addition, AsA and GSH participate in the AsA–GSH cycle and contribute to non-enzymatic ROS scavenging [33,34]. In this study, MAP treatment maintained higher SOD, CAT, and APX activities and delayed the depletion of AsA and GSH. MAP also maintained higher DPPH radical scavenging capacity and reducing power, indicating improved antioxidant capacity. These responses are consistent with previous findings that MAP enhanced antioxidant enzyme activities and delayed the decline of AsA and GSH in pomegranate peel [35]. Preservation treatments that regulate ROS metabolism have also been shown to maintain antioxidant capacity and membrane integrity in fresh produce [11,14]. Therefore, MAP alleviated oxidative stress in *P. pulmonarius* by activating enzymatic and non-enzymatic antioxidant systems, thereby contributing to quality maintenance.

### 4.4. Effects of Membrane Lipid Metabolism on the Quality of MAP-Treated P. pulmonarius Under Simulated Logistics

Cell membrane integrity is essential for maintaining cellular homeostasis and delaying postharvest senescence. Increased membrane permeability reflects membrane structural damage and is closely associated with tissue softening, browning, and quality deterioration during storage [24]. In this study, membrane permeability increased continuously in both groups, but the increase was significantly lower in the MAP group. This indicates that MAP effectively maintained membrane integrity under simulated logistics conditions.

Membrane lipid degradation is closely related to the activities of lipase, LOX, and PLD. During storage, PLD can hydrolyze membrane phospholipids, lipase can release free fatty acids, and LOX can catalyze the peroxidation of unsaturated fatty acids, eventually leading to membrane destabilization and oxidative damage. In the present study, the activities of lipase, LOX, and PLD increased during storage, whereas MAP treatment significantly suppressed these increases. Correlation analysis showed that the activities of these enzymes were positively correlated with membrane permeability, suggesting that enhanced membrane lipid degradation contributed to membrane damage.

Phospholipid components are important structural and signaling molecules in cell membranes. PC and PI contribute to membrane integrity and signal regulation, whereas PA is associated with stress responses and phospholipid turnover [28]. In this study, PC and PI decreased while PA increased during storage. MAP treatment delayed the degradation of PC and PI and inhibited PA accumulation, indicating reduced phospholipid hydrolysis. Similar changes in phospholipid metabolism have been reported in harvested *Hericium erinaceus*, where preservation treatment regulated PC, PI, and PA levels and alleviated fruit-body softening [36].

Fatty acid composition also affects membrane fluidity and stability. A higher proportion of unsaturated fatty acids helps maintain membrane fluidity, whereas the decline in USFAs and increase in SFAs are associated with membrane rigidification and senescence [12,37]. In this study, USFAs, including oleic acid, linoleic acid, and linolenic acid, decreased during storage, whereas SFAs increased. However, MAP treatment delayed USFA degradation and maintained higher U/S and IUFA values. These results are consistent with previous findings that preservation treatments can maintain mushroom quality by regulating membrane lipid metabolism [12,37]. Overall, these results indicate that MAP helped maintain membrane integrity by suppressing lipid-degrading enzyme activities, delaying phospholipid degradation, maintaining fatty acid unsaturation, and reducing membrane permeability. Together with the observed reductions in weight loss and browning and the maintenance of sensory quality, the preservation of membrane stability may partly explain the delayed postharvest deterioration of *P. pulmonarius* under simulated logistics temperature fluctuations.

## 5. Conclusions

In conclusion, this study demonstrates that modified atmosphere packaging (MAP) is an effective strategy for maintaining the postharvest quality of *P. pulmonarius* under simulated logistics temperature fluctuations. The optimal MAP condition was identified as 5% O_2_ and 20% CO_2_ with EVOH film. Under this condition, MAP-treated samples maintained higher sensory quality, lower weight loss, reduced browning, and higher total phenolic and flavonoid contents than the control, indicating that MAP effectively delayed quality deterioration during simulated logistics and subsequent storage.

At the physiological and biochemical levels, MAP alleviated oxidative stress and maintained membrane lipid stability. MAP treatment inhibited O_2_^.^^−^ and MDA accumulation, enhanced SOD, CAT, and APX activities, and delayed the depletion of AsA and GSH. Meanwhile, MAP reduced membrane permeability, suppressed lipase, LOX, and PLD activities, delayed PC and PI degradation, inhibited PA accumulation, and maintained higher unsaturated fatty acid levels, U/S, and IUFA values. These results suggest that MAP delays the postharvest senescence of *P. pulmonarius* by maintaining ROS homeostasis and membrane lipid stability.

Overall, the optimized MAP treatment, consisting of 5% O_2_ + 20% CO_2_ with EVOH film, improved the quality maintenance of fresh *P. pulmonarius* during the tested 5-day simulated logistics and cold storage period. However, because the EVOH film had a very low OTR, the applicability of this system to longer commercial storage should be further verified to avoid potential O_2_ depletion, anaerobic metabolism, and off-odor formation.

## Figures and Tables

**Table 1:** Factors and levels of the orthogonal experimental design.

Level	Oxygen Concentration (A)	Carbon Dioxide Concentration (B)	Packaging Film (C)
1	0%	10%	EVOH film
2	5%	20%	PET/PE film
3	10%	30%	PA/PE film

**Table 2:** Sensory scoring criteria for postharvest quality evaluation of *P. pulmonarius*.

Evaluation Item	25–19	19–14	13–8	7–0
Color	Normal color with gloss	Slightly yellow with reduced gloss	Yellow with dull gloss	Brown with no gloss
Odor	Normal mushroom aroma	Slight off-odor and weak alcoholic odor	Obvious off-odor and alcoholic odor	Severe off-odor and strong alcoholic odor
Morphology	Intact pileus	Slight cracking of pileus	Severe cracking of pileus	Broken pileus
Texture	Good elasticity	Moderate elasticity	Soft texture	Broken pileus

**Table 3:** Orthogonal experimental results.

Treatment No.	A	B	C	Sensory Score	BI	MDA Content (nmol/g)
1	1	1	1	78.75 ± 0.43	43.38 ± 0.65	20.06 ± 0.14
2	1	2	2	77.88 ± 0.93	42.10 ± 0.88	20.26 ± 0.29
3	1	3	3	75.88 ± 0.78	41.59 ± 1.40	21.10 ± 0.33
4	2	1	2	81.88 ± 0.66	44.15 ± 0.71	20.11 ± 0.16
5	2	2	3	83.25 ± 0.97	42.67 ± 0.69	19.02 ± 0.20
6	2	3	1	79.88 ± 1.17	41.75 ± 0.81	21.07 ± 0.27
7	3	1	3	80.38 ± 0.86	46.68 ± 0.83	22.36 ± 0.17
8	3	2	1	81.13 ± 0.87	45.44 ± 0.31	21.31 ± 0.33
9	3	3	2	78.13 ± 0.97	44.17 ± 0.54	24.03 ± 0.32

**Table 4:** Range analysis of the orthogonal experiment.

Index	Factor	K1	K2	K3	k1	k2	k3	R
Sensory score	A	232.50	244.88	239.88	77.50	81.63	79.96	4.13
	B	241.13	242.63	233.50	80.38	80.88	77.83	3.04
	C	240.13	239.75	237.38	80.04	79.92	79.13	0.92
BI	A	127.07	128.57	136.29	42.36	42.86	45.43	3.07
	B	134.21	130.20	127.51	44.74	43.40	42.50	2.23
	C	130.57	130.93	130.42	43.52	43.64	43.47	0.17
MDA content	A	61.42	60.20	67.70	20.47	20.07	22.57	2.50
	B	62.52	60.60	66.20	20.84	20.20	22.07	1.87
	C	62.44	62.49	64.40	20.81	20.83	21.47	0.65

**Table 5:** Analysis of variance of the orthogonal experiment.

Source	df	SS of Sensory Score	MS of Sensory Score	F	SS of BI	MS of BI	F	SS of MDA	MS of MDA	F
A	2	25.82	12.91	83.53	16.31	8.16	104.84	10.79	5.40	47.34
B	2	15.94	7.97	51.59	7.58	3.79	48.69	5.41	2.71	23.73
C	2	1.49	0.74	4.80	0.05	0.02	0.29	0.83	0.42	3.65
Error	2	0.31	0.34		0.16	0.08		0.23	0.11	
Total	8	43.55			24.09			17.27		

Note: F_0.05_(2,2) = 19; F_0.01_(2,2) = 99.

**Table 6:** Validation test results of two candidate MAP combinations for *P. pulmonarius* under simulated logistics temperature fluctuations.

Indicator	Combination 1	Combination 2	Significance
Sensory score	83.88 ± 0.74	77.25 ± 0.91	**
Weight loss (%)	3.03 ± 0.08	3.19 ± 0.21	ns
Hardness (gf)	428.95 ± 27.03	315.46 ± 31.34	**
Total soluble solids (%)	2.67 ± 0.09	2.59 ± 0.15	ns
Soluble protein content (mg/g)	5.47 ± 0.16	4.92 ± 0.17	*
Total sugar content (mg/g)	26.10 ± 0.10	23.92 ± 0.24	**
Browning index	41.79 ± 0.53	39.80 ± 0.57	*
MDA content (nmol/g)	19.20 ± 0.35	20.92 ± 0.11	**
Membrane permeability (%)	22.87 ± 0.32	32.32 ± 0.61	**

Note: Combination 1: 5% O_2_ + 20% CO_2_ with EVOH film; Combination 2: 0% O_2_ + 30% CO_2_ with PA/PE film. ns, non-significant; *, *p* < 0.05; **, *p* < 0.01.

**Table 7:** Relative contents of membrane fatty acid components in fresh *P. pulmonarius*.

Category	Fatty Acid	Relative Content (%)
C_14:0_	Myristic acid	0.30 ± 0.01
C_16:0_	Palmitic acid	7.99 ± 0.03
C_18:0_	Stearic acid	1.04 ± 0.02
C_18:1_	Oleic acid	10.60 ± 0.24
C_18:2_	Linoleic acid	78.44 ± 0.08
C_18:3_	Linolenic acid	0.40 ± 0.02
C_20:0_	Arachidic acid	0.42 ± 0.02
C_24:0_	Lignoceric acid	0.60 ± 0.00

## Data Availability

The original contributions presented in the study are included in the article; further inquiries can be directed to the corresponding author.
